# Parasympathetic Stimuli on Bronchial and Cardiovascular Systems in Humans

**DOI:** 10.1371/journal.pone.0127697

**Published:** 2015-06-05

**Authors:** Emanuela Zannin, Riccardo Pellegrino, Alessandro Di Toro, Andrea Antonelli, Raffaele L. Dellacà, Luciano Bernardi

**Affiliations:** 1 Dipartimento di Elettronica, Informazione e Bioingegneria, Politecnico di Milano, Milano, Italy; 2 Allergologia e Fisiopatologia Respiratoria, ASO S. Croce e Carle, Cuneo, Italy; 3 Dipartimento di Medicina Interna, Università di Pavia e IRCCS Policlinico S. Matteo, Pavia, Italy; 4 Folkhälsan Institute of Genetics, Folkhälsan Research Center, Biomedicum Helsinki, Helsinki, Finland; Bascom Palmer Eye Institute, University of Miami School of Medicine;, UNITED STATES

## Abstract

**Background:**

It is not known whether parasympathetic outflow simultaneously acts on bronchial tone and cardiovascular system waxing and waning both systems in parallel, or, alternatively, whether the regulation is more dependent on local factors and therefore independent on each system. The aim of this study was to evaluate the simultaneous effect of different kinds of stimulations, all associated with parasympathetic activation, on bronchomotor tone and cardiovascular autonomic regulation.

**Methods:**

Respiratory system resistance (Rrs, forced oscillation technique) and cardio-vascular activity (heart rate, oxygen saturation, tissue oxygenation index, blood pressure) were assessed in 13 volunteers at baseline and during a series of parasympathetic stimuli: O_2_ inhalation, stimulation of the carotid sinus baroreceptors by neck suction, slow breathing, and inhalation of methacholine.

**Results:**

Pure cholinergic stimuli, like O_2_ inhalation and baroreceptors stimulation, caused an increase in Rrs and a reduction in heart rate and blood pressure. Slow breathing led to bradycardia and hypotension, without significant changes in Rrs. However slow breathing was associated with deep inhalations, and Rrs evaluated at the baseline lung volumes was significantly increased, suggesting that the large tidal volumes reversed the airways narrowing effect of parasympathetic activation. Finally inhaled methacholine caused marked airway narrowing, while the cardiovascular variables were unaffected, presumably because of the sympathetic activity triggered in response to hypoxemia.

**Conclusions:**

All parasympathetic stimuli affected bronchial tone and moderately affected also the cardiovascular system. However the response differed depending on the nature of the stimulus. Slow breathing was associated with large tidal volumes that reversed the airways narrowing effect of parasympathetic activation.

## Introduction

In humans, airway smooth muscle (ASM) tone is largely determined by parasympathetic cholinergic control, which is operated by the vagus that innervates the large airways [1].

The central autonomic control of the ASMs tone is a complex and interconnected system with multiple parallel pathways that contribute to regulate the parasympathetic cholinergic outflow in multiple organs and systems. Dysfunction or dysregulation of the autonomic control of ASMs contributes to the pathogenesis of asthma and chronic obstructive pulmonary disease, and may also produce the respiratory symptoms associated with cardiovascular diseases [2–5].

A comprehensive knowledge of the mechanisms regulating ASMs tone would be crucial for a better understanding and treatment of obstructive pulmonary diseases. However, there are still tremendous gaps in our understanding of airway neural control, even in the healthy lung. One of these gaps is related to the central control of autonomic tone and to the integration of different afferent inputs.

Due to the limited availability of methods capable of simultaneously assessing ASMs tone and cardiovascular regulation, the interaction between these two systems remains poorly studied. In particular, it is not known whether parasympathetic activation acts on the bronchial tone and cardiovascular system in parallel, or, alternatively, whether the regulation of the two systems is more determined by local factors acting on each system independently. Moreover it is unclear whether all stimuli associated with parasympathetic activation have similar effects or whether different stimuli might have different effects according to their specific nature. We hypothesized that interventions that stimulate the cardiovascular and respiratory systems through parasympathetic activation would cause both cardiovascular depression and airway narrowing, while interventions associated with a selective direct stimulation of either system would produce independent responses.

To this aim we evaluated the simultaneous effects of different kinds of stimulations, all associated to parasympathetic activation, on bronchomotor tone and cardiovascular autonomic regulation to test whether the control of the cardiovascular and respiratory systems acts separately or in parallel, according to their different needs. In particular we evaluated the effect of neck suction, oxygen inhalation, slow breathing (common in Yoga and other similar practices) and methacoline (Mch) administration in a group of healthy volunteers. Neck suction is a pure carotid baroreceptors stimulation [6–9]. Oxygen inhalation increases cardiac parasympathetic activity [10–12], but may also alter gas exchange and consequently affect ventilation. Slow breathing increases the vagal arm of the cardiac baroreflex [13–16], but also modifies gas exchange [17,18] and, through the increase in tidal volume, may also affect bronchial tone [19]. Finally, inhaled MCh induces airway narrowing as a result of parasympathetic activation, but may also activate sympathetic reflexes in other systems due to the associated hypoxemia [20].

## Materials and Methods

### Subjects

The study was conducted in 13 healthy subjects whose characteristics are reported in Table 1. None of them was taking any treatment at the time of the study. The study was approved by the Ethical Committee of the S. Croce and Carle Hospital (FPResp 13, 26/7/13, Cuneo, Italy), and written informed consent was obtained from each subject prior to the study.

**Table 1:** Subjects’ anthropometric characteristics and main lung functional data.

Sex, M/F	8/6
Age, yr	41±14
Height, cm	172±8
BMI, kg·m^-2^	22±3
FEV_1_, % of predicted	108±9
FVC, % of predicted	114±10
FEV_1_/FVC, %	80 ± 9

BMI, body mass index; FEV_1_, forced expiratory volume in 1 s; FVC, forced vital capacity. Data are mean ± SD.

### Pre-study evaluations

The subjects underwent a spirometric test and methacholine challenge to identify the dose causing a 20% decrease of FEV_1_ (PD20FEV_1_).

### Measurements

Detailed description of methodology and data analysis is reported in S1 Supporting Information.

Electrocardiogram was measured by placing three electrodes on the patient's anterior chest wall. Oxygen saturation (SaO_2_) was measured at the finger with a pulse oxymeter and expired carbon dioxide (CO_2_) by a capnograph. Oxygenation perfusion at the tissue level was estimated at the left forearm by a Near Infrared Spectroscope. Values are expressed as tissue oxygen saturation index (TO_2_I). Continuous noninvasive arterial blood pressure was monitored via cuffs positioned on the middle finger of the right arm held at the heart level. All signals were simultaneously acquired at 400 Hz on a Macintosh laptop (Apple, Coupertino, CA) with a 12 channel acquisition system.

Airway mechanics was measured by multiple frequency forced oscillation technique (FOT) at 5, 11, and 19 Hz [21–23]. Respiratory system resistance was computed by a least squares algorithm [24,25] at 5 Hz (R_5_) and 19 Hz (R_19_).

### Parasympathetic stimuli

Oxygen (O_2_) inhalation was obtained by breathing supplemental O_2_ for 11 min. O_2_ supplementation was obtained by mixing 5 L·min^-1^ of air and 5 L·min^-1^ O_2_ using a douglas bag. Therefore the percent of O_2_ in the inspired gas was 60%. Measurements were taken during the last 5 min.

Baroreceptors stimulation was obtained by sinusoidal suction applied to a lead collar positioned around the neck by a vacuum system via a computer-controlled valve which produced a controlled suction loss [26]. Breathing was paced at a fixed breathing frequency of 15 breath·min^-1^ imposed by a metronome in order to avoid any entraining effect on breathing by the frequency of neck suction. Measurements were taken during 2 min of sinusoidal suction from 0 to -30 mmHg at 0.1Hz, and 2 min at 0.2 Hz [9]. The control condition was represented by tidal breathing at 15 breath·min^-1^. Slow deep breathing (SB) was obtained by imposing a fixed frequency of 6 breath·min^-1^ (5 sec inhalation, 5 sec exhalation) and leaving the subjects regulate their tidal volume as needed for 2 min, during which measurements were taken.

MCh challenge was performed by administering the PD20FEV1 estimated in the pre-study day. Two min after the end of the inhalation, measurements were taken during 2 min of spontaneous breathing.

### Protocol

The study was conducted in the sitting position. All measurements described above were performed at baseline conditions for 4 min, and then in random order during O_2_ breathing, slow deep breathing, and neck suction at 0.1 and 0.2 Hz. The bronchial challenge was always performed at the end of study to avoid the long-lasting effects of the constrictor agent on airway tone control.

### Data Analysis

The effects of the parasympathetic activity on the cardiovascular system were estimated from the RR interval, systolic (SBP) and diastolic blood pressures (DBP) and baroreflex sensitivity (BRS). The latter was the mean value computed from seven different tests [27, 28].

Average heart rate (HR) was calculated for each sequence. Heart rate variability was analyzed in terms of SD of each RR sequence and in terms of root mean square of successive differences (RMSSD). At all conditions BRS, HR and HR variability were compared with resting baseline values.

The parasympathetic effects of neck suction were estimated from the power spectra of the RR interval and blood pressure signals evaluated at 0.1 Hz, when the neck suction was timed at 0.1 Hz, and from the power spectra at 0.2 Hz, when the neck suction was timed at 0.2 Hz. Since breathing frequency was fixed at 15 breath·min^-1^, this approach allowed separating the effects of ventilation, which are more complex than the simple baroreflex effect, from the pure baroreflex stimulus within the respiratory range.

**Fig 1:**
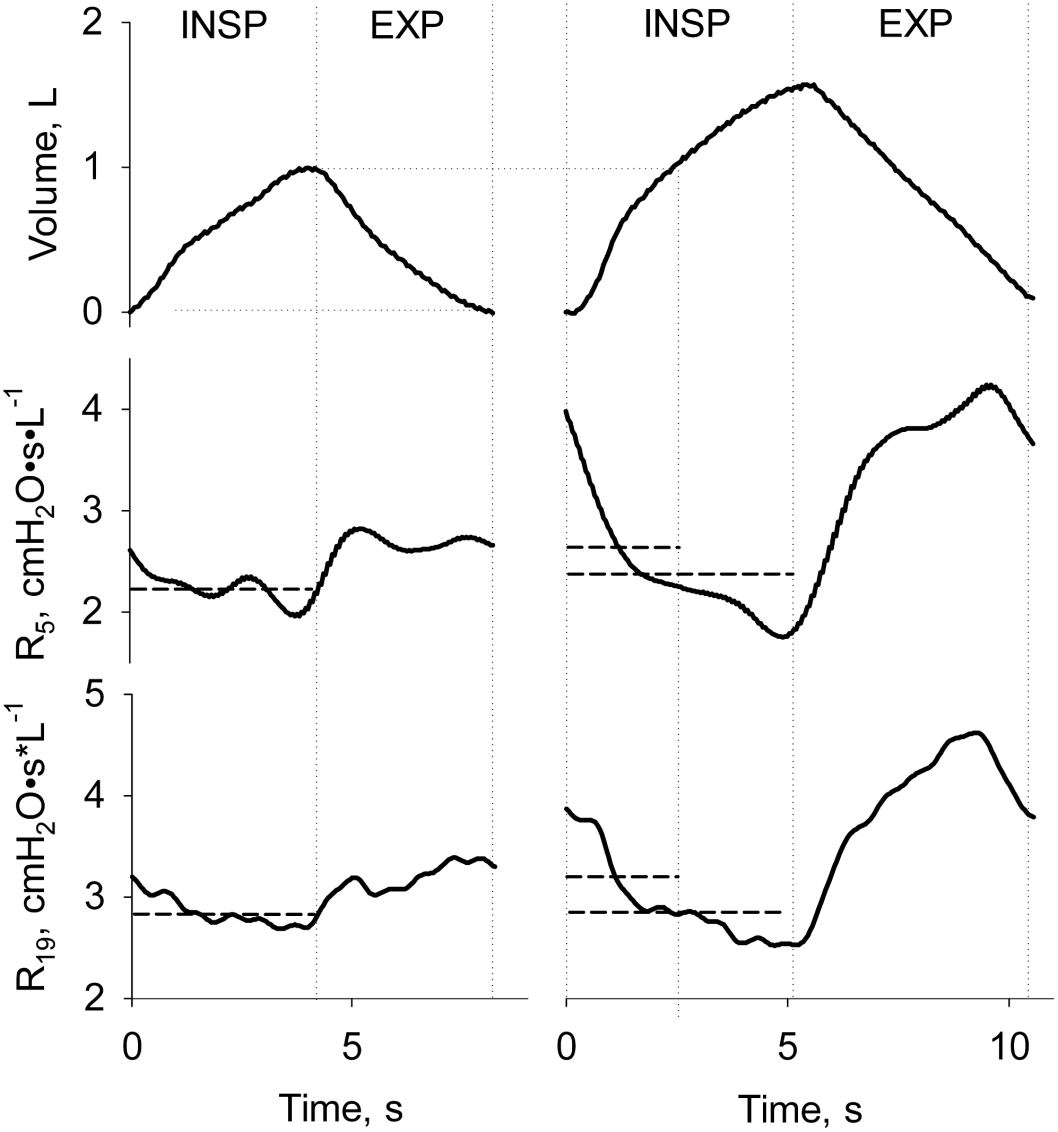
Typical example of measurements of airway resistance during tidal (left panels) and slow deep breathing experimental conditions (right panels). Tidal volume and resistance at 5 (R_5_) and 19 Hz (R_19_) are shown as a function of time in the upper, mid, and lower panels, respectively. Direction of the inspiratory and expiratory phases is indicated. Vertical grey dotted lines from left to right identify the beginning and the end of tidal breathing as well as mid volume for the large breath. Horizontal gray continuous lines are average R_5_ and R_19_ measured over the whole inspiration or the portion of volume corresponding to tidal breathing. The latter are named R_5-IsoVol_ and R_19-IsoVol_ in the text.

R_5_ and R_19_, evaluated during tidal inspiration, were taken as indexes of total and central airways size, respectively [21]. For SB conditions R_5_ and R_19_ were also measured over the segment of inspiration corresponding to control tidal volume (R_5-IsoVol_ and R_19-IsoVol_), as shown in Fig 1. This allowed separating the effects of the parasympathetic stimuli from depth of breathing on bronchomotor tone.

The effects of the different interventions on gas exchange and O_2_ delivery to the tissues were estimated from the changes in SaO_2_ and TO_2_I.

### Statistical analysis

Differences between groups were tested for statistical significance by a one-way analysis of variance (ANOVA) with Holm-Sidak post-hoc test for multiple-comparisons or paired t-test wherever applicable. Values of p<0.05 were considered statistically significant. Data are presented as mean ± standard deviation (SD).

## Results

The main anthropometric and functional parameters of the subjects are shown in Table 1. PD20FEV1 was 2400 μg for all subjects but one who responded at 200 μg.

### Effect of O_2_ inhalation

Supplemental O_2_ caused a marked increase in RR interval (p<0.001), a slight decrease in SBP (p = 0.051) and no relevant changes in BRS and RMSSD. R_5_ and R_19_ slightly but significantly increased by similar amount. Minute Ventilation ($\dot{V}$_E_) slightly but significantly decreased (p = 0.020). SaO_2_ but not TO_2_I significantly increased (p<0.001). Collectively, these findings suggest that supplemental O_2_ caused mild depression of the cardiovascular system and central airways narrowing. The main data are reported in Table 2.

**Table 2:** Main cardiovascular and FOT parameters at control (room air) and during O_2_ (5 L∙min^-1^) breathing conditions.

	Control (Room air)	Quiet breathing (O_2_ 5 L∙min^-1^)	Paired t-test
Mean RR interval, ms	814 (109)	872 (98)	< 0.001
RR-SD, ms	50 (28)	44 (24)	0.204
RMSSD, ms	47 (26)	40 (18)	0.172
SBP, mm Hg	133 (26)	121 (25)	0.051
DBP, mm Hg	75 (13)	72 (15)	0.173
BRS, ms∙mmHg-1	12.2 (6.8)	13.0 (7.7)	0.582
SaO_2_, %	98 (1)	99 (1)	< 0.001
TO_2_I, %	65 (10)	65 (12)	0.959
R_5_, cm H_2_O∙s∙L^-1^	1.70 (0.55)	2.12 (0.82)	0.006
R_19_, cm H_2_O∙s∙L^-1^	1.87 (0.61)	2.45 (0.88)	< 0.001
V_T_, L	0.93 (0.17)	0.76 (0.17)	0.209
$\dot{V}$_E_, L∙min^-1^	9.98 (2.01)	8.06 (2.60)	0.020
CO_2-ET_, mm Hg	40.5 (4.02)	34.69 (5.35)	< 0.001

RR interval: interval between two RR wave peaks; RR-SD: standard deviation of RR interval; SBP and DBP: systolic and diastolic pressures, respectively; SaO_2_: oxygen saturation; TO_2_I: Tissue Oxygen Index; R_5_ and R_19_, inspiratory resistance at 5 and 19 Hz, respectively; V_T_: tidal volume; $\dot{V}$_E_: minute ventilation; CO_2-ET_: carbon dioxide end-tidal. Data are mean ± SD.

### Effect of neck suction

Neck suction at 0.1 Hz and at 0.2 Hz significantly increased the oscillations in the RR interval power spectrum at 0.1 Hz (p<0.001) and at 0.2 Hz (p = 0.05). Neck suction at both frequencies was associated with an increase in both R_5_ and R_19_, though not statistically significant. Tidal Volume (Vt), $\dot{V}$_E_, SaO_2_ and TO_2_I remained unmodified. Data are reported in Table 3.

**Table 3:** Main cardiovascular and FOT parameters at baseline (room air) and neck suction at 0.1 and 0.2 Hz. For all conditions breathing frequency is 15 breaths•min^-1^.

	Control (15 bpm)	Neck suction (0.1 Hz)	Neck suction (0.2 Hz)	Paired t-test or ANOVA
RR0.1 Hz, ln-Power	5.96 (0.65)	7.02 (0.92)	-	< 0.001
RR0.2 Hz, ln-Power	5.98 (1.09)	-	6.37 (1.10)	0.042
Mean RR interval, ms	769 (102)*	792 (89)*	782 (93)	0.031
RR-SD, ms	31 (13)*	47 (19)* §	36 (13)§	< 0.001
RMSSD, ms	32 (13)*	44 (15)* §	36 (12)§	0.003
SBP, mm Hg	124 (23)	126 (24)	123 (19)	0.711
DBP, mm Hg	69 (17)	72 (14)	70 (15)	0.593
SaO_2_, %	98 (1)	98 (1)	98 (1)	0.937
TO_2_I, %	64 (8)	64 (6)	64 (6)	0.721
R_5_, cm H_2_O∙s∙L^-1^	2.00 (0.68)	2.18 (0.62)	2.24 (0.67)	0.059
R19, cm H_2_O∙s∙L^-1^	2.22 (0.76)	2.35 (0.63)	2.43 (0.66)	0.128
V_T_, L	1.23 (0.51)	1.28 (0.44)	1.30 (0.49)	0.424
$\dot{V}$_E_ L∙min^-1^	18.74 (7.59)	19.09 (6.45)	8.06 (7.24)	0.451
CO_2-ET_, mm Hg	33.51 (3.81)¶	32.65 (3.49)§	30.70 (4.71)§ ¶	< 0.001

RR interval: interval between two RR wave peaks; RR-SD: standard deviation of RR interval; SBP and DBP: systolic and diastolic pressures, respectively; SaO_2_: oxygen saturation; TO_2_I: Tissue Oxygen Index; R_5_ and R_19_, inspiratory resistance at 5 and 19 Hz, respectively; V_T_: tidal volume; $\dot{V}$_E_: minute ventilation; CO_2-ET_: carbon dioxide end-tidal. Data are mean ± SD. RR0.1 Hz and RR0.2 Hz are expressed as natural logarithm of power spectra of RR interval at 0.1 and 0.2 Hz, respectively. t-test was used to compare RR0.1 between control and Neck suction (0.1 Hz), and to compare RR0.2 between control and Neck suction (0.2 Hz). ANOVA was used for all the other parameters that were compared for the three conditions.

*: p<0.05 between Neck suction (0.1 Hz) and Control by post-hoc analysis

^¶^: p<0.05 between Neck suction (0.2 Hz) and Control by post-hoc analysis

^§^: p<0.05 between Neck suction (0.1 Hz) and Neck suction (0.2 Hz) by post-hoc analysis.

### Effect of slow deep breathing

Slow breathing was achieved by doubling Vt (p<0.001). $\dot{V}$_E_ remained constant. This was associated with an increase in RR-SD (p<0.001) but no significant changes in RR, BRS, RMSSD or blood pressure. R_5-IsoVol_ and R_19-Iso-Vol_ increased by similar amount (p<0.001 for both). These findings suggest mild depression of the cardiovascular system and central airways narrowing. However, R_5_ and R_19_ remained similar to baseline conditions. SaO_2_ and TO_2_I slightly but significantly increased (p<0.05 for both). Data are reported in Table 4.

**Table 4:** Main cardiovascular and respiratory and FOT parameters during slow breathing conditions.

	Control (Room air)	6 breaths∙min^-1^	Paired t-test
Mean RR interval, ms	814 (109)	814 (94)	0.990
RR-SD, ms	50 (28)	79 (25)	< 0.001
RMSSD, ms	47 (26)	48 (16)	0.817
SBP, mm Hg	133 (26)	132 (24)	0.483
DBP, mm Hg	75 (13)	71 (17)	0.133
BRS, ms∙mmHg-1	12.2 (6.8)	13.7 (7.4)	0.263
SaO_2_, %	98 (1)	98 (1)	0.017
TO_2_I, %	65 (10)	66 (10)	0.053
R_5_, cm H_2_O∙s∙L^-1^	1.70 (0.55)	1.85 (0.50)	0.579
R_5-IsoVol_, cm H_2_O∙s∙L^-1^	-	2.58 (0.82)	< 0.001
R_19_, cm H_2_O∙s∙L^-1^	1.87 (0.61)	2.09 (0.59)	0.443
R_19-IsoVol_, cm H_2_O∙s∙L^-1^	-	2.59 (0.78)	< 0.001
V_T_, L	0.93 (0.17)	1.92 (0.60)	< 0.001
$\dot{V}$_E_, L∙min-1	9.98 (2.01)	11.21 (3.49)	0.110
C_O2-ET_, mm Hg	40.5 (4.02)	38.82 (3.48)	0.403

RR interval: interval between two RR wave peaks; RR-SD: standard deviation of RR interval; SBP and DBP: systolic and diastolic pressures, respectively; SaO_2_: oxygen saturation; TO_2_I: Tissue Oxygen Index; R_5_ and R_19_, inspiratory resistance at 5 and 19 Hz, respectively; V_T_: tidal volume; $\dot{V}$_E_: minute ventilation; CO_2-ET_: carbon dioxide end-tidal. Data are mean ± SD. R_5-IsoVol_ and R_19-IsoVol_: resistance at 5 and 19 Hz measured at control lung volume.

### Effect of inhaling metacholine

Inhaling MCh caused a significant increase in bronchial tone as documented by remarkable increments in R_5_ and R_19_ (p<0.001 for both). The increase of the former over the latter (p<0.001) is consistent with heterogeneous distribution of airway narrowing across the lungs. In contrast, all cardiovascular parameters remained unmodified. Vt slightly but significantly decreased (p<0.001). $\dot{V}$_E_ tended to decrease. SaO_2_ and TO_2_I significantly decreased (p<0.001 for both). Data are reported in Table 5.

**Table 5:** Main cardiovascular and FOT parameters at baseline (room air) and after methacholine (MCh).

	Control	Mch	Paired t-test
Mean RR interval, ms	814 (109)	798 (125)	0.189
RR-SD, ms	50 (28)	39 (24)	0.040
RMSSD, ms	47 (26)	38 (20)	0.069
SBP, mm Hg	133 (26)	130 (19)	0.709
DBP, mm Hg	75 (13)	74 (15)	0.892
BRS, ms∙mmHg^-1^	12.2 (6.8)	10.5 (7.8)	0.184
SaO_2_, %	98 (1)	94 (3)	0.001
TO_2_I, %	65 (10)	61 (11)	< 0.001
R_5_, cm H_2_O∙s∙L^-1^	1.70 (0.55)	4.57 (1.26)	< 0.001
R_19_, cm H_2_O∙s∙L^-1^	1.87 (0.61)	3.47 (0.93)	< 0.001
V_T_, L	0.93 (0.17)	0.64 (0.14)	0.001
$\dot{V}$_E_, L∙min^-1^	9.98 (2.01)	8.26 (2.51)	0.090
CO_2-ET_, mm Hg	40.5 (4.02)	34.46 (3.51)	< 0.001

RR interval: interval between two RR wave peaks; RR-SD: standard deviation of RR interval; SBP and DBP: systolic and diastolic pressures, respectively; SaO_2_: oxygen saturation; TO_2_I: Tissue Oxygen Index; R_5_ and R_19_, inspiratory resistance at 5 and 19 Hz, respectively; V_T_: tidal volume; $\dot{V}$_E_: minute ventilation; CO_2-ET_: carbon dioxide end-tidal. Data are mean ± SD.

## Discussion

We evaluated the effects of different interventions, expected to activate parasympathetic system, on bronchial tone and cardiovascular regulation. For all interventions the observed cardiovascular response was in agreement with previously published works [7,13,15,29–33]. This was considered as a proof that all interventions actually produced a parasympathetic activation. Moreover, all the interventions were also associated with tonic activation of the ASMs, but with important differences in terms of change in airways resistance among different stimuli. This result is consistent with a different balance between the tonic activation mediated by the parasympathetic system and local factors such as the mechanical action operated by ventilation on the airways walls. On the other hand, inhaled MCh caused airway narrowing but not cardiovascular depression.

The simultaneous assessment of the cardiovascular regulation and bronchial tone during different parasympathetic stimulations was possible thanks to the use of FOT for the evaluation of bronchial tone. This technique is very sensitive to changes in airway caliber [34–36] and allows the assessment of the respiratory system resistance without altering the breathing pattern, including spontaneous, paced and slow breathing. Moreover, using a within-breath approach, respiratory resistance can be assessed at any lung volume, allowing the discrimination between the contribution of ASMs tone and lung volume dependence to changes in lung mechanics. Finally FOT does not require the execution of special respiratory maneuvers, like deep inhalation in spirometry, which may themselves counteract the effect of bronchocontricting interventions [19].

### Determinants of ASMs tone

The most relevant determinant of baseline cholinergic tone of ASMs is ventilation: increasing or decreasing respiratory rate increases and decreases baseline cholinergic tone, respectively. Ventilation is also the primary antagonist of ASMs tone with tidal breaths cyclically tethering the airways walls counterbalancing the effects of parasympathetic activation [37]. Within tidal breathing range, the tonic activation mediated by the parasympathetic system prevails over the mechanical action operated by ventilation, keeping the airways slightly contracted [38]. Thanks to the presence of a basal cholinergic tone, contractions and relaxations can be mediated reflexively [39–41]. Reflex-mediated contractions of ASMs can be initiated by different stimuli delivered both to the airways and lungs as well as to the heart, arterial baroreceptors and chemoreceptors [39–41]. Smooth muscle contraction can evoke a further reflex-mediated contraction of the ASMs. Therefore, bronchoconstrictors evoke bronchospasm at least in part by initiating parasympathetic-cholinergic reflexes [38].

### Specific response to the different parasympathetic stimuli

Supplemental O_2_ is known to evoke vagal reflexes in disease conditions, and to a lesser extent in healthy subjects. This is due both to local and systemic factors. Vasoconstriction, due to the direct effect of O_2_ and O_2_ reactive species on the vascular smooth muscles, is thought to be the primary response [42]. Vasoconstriction is associated with hypertension, while bradycardia is a secondary effect associated with the baroreflex activation. Moreover, at a lung level an excess of free radicals presumably stimulates the parasympathetic lung afferents [11,43]. In our study the bronchomotor and cardiovascular responses to O_2_ inhalation are consistent with parasympathetic activation. The increase in both R_5_ and R_19_ suggests that narrowing involved the central airways where the cholinergic receptors are mostly expressed. Heart rate decreased, blood pressure slightly decreased, though this was not associated with an increase in BRS. This result combined with the lack of increase in TO_2_I despite the increase in SaO_2_, would suggest that O_2_ induced peripheral vasoconstriction, preventing O_2_ from flowing to the tissues.

Neck suction is a “pure” autonomic stimulus that is not associated with any hemodynamic effect other than reflex [7]. The neck suction at 0.2 Hz (close to but different from the respiratory frequency at 0.25 Hz) causes selective vagal modulation, whereas at 0.1 Hz causes both vagal and sympathetic modulation [7,44]. The efficacy of the technique in causing selective vagal modulation at the cardiovascular level in our study is documented by a prevalent increase in the RR interval variability with no significant changes in systolic and diastolic blood pressure. Differently from supplemental O_2_, sinusoidal neck suction induces a modulation in autonomic function rather than steady-state changes. The mean values of most variables remained unchanged while RR interval fluctuations coherent with the frequency of neck suction increased. At the bronchial level, the neck suction was associated with a significant increase in R_5_ and with only a slight not significant increase in R_19_, suggesting that narrowing occurred at a slightly more peripheral site than with O_2_ inhalation.

Breathing at 6 cycles/min, RR fluctuations merge at the rate of respiration and their amplitude increases relative to blood pressure changes, enhancing the vagal arm of baroreflex [14–16]. At lung level the increase in tidal volume derived by slow breathing directly increases vagal activity and reduces the sympathetic activity via the Hering–Breuer reflex [13], which in turn reduces the chemoreflex sensitivity and thus might further enhance the baroreflex. Additionally, slow breathing increases O_2_ saturation as a result of the decrease of physiologic dead space and improved ventilation/perfusion matching [18]. In the present study slow breathing led to cardiovascular inhibition and airway narrowing, as suggested by an increase in BRS and R_5-IsoVol_ and R_19-Iso-Vol_. However slow breathing was associated with large tidal volumes that reversed the airways narrowing effect of parasympathetic activation due to the well known inverse relationship between lung volume and airway size [45], that leads to direct bronchodilatation at large tidal volumes [19]. Therefore, slow breathing *per se* caused some constrictive response (suggested by the increase in R_5-IsoVol_ and R_19-IsoVol_) but this was ablated by larger tidal volumes.

Inhaling MCh induced marked airway narrowing (R_5_ and R_19_) and increase in dyspnea [38]. Despite airway narrowing, none of the cardiovascular parameters exhibited significant changes relative to control. We speculate that hypoxemia due to airway narrowing elicited sympathetic reflexes [20] which counteracted the cardiovascular depressive effects of the vagal stimulation.

### Clinical implications

Yoga, Zen and physical training are proved to be beneficial for the cardiovascular function [46–49], as they modify the autonomic balance toward a parasympathetic predominance and they strengthen baroreflex, resulting in reduced HR, reduced blood pressure, increased HR variability and increased baroreflex sensitivity, which in turn reduce the load on the heart and cardiovascular system in general. The results of the present study confirm that, and help understand why, these natural stimuli lead to favorable cardiovascular effects without hampering airway function.

## Conclusions

All parasympathetic stimuli tested in the present study affected the bronchial tone and moderately affected also the cardiovascular system. When the stimuli were carried by complex interventions that modified breathing pattern or gas exchange the responses were ablated at either level. Slow breathing modulated the cardiovascular activity but left bronchial tone unmodified, likely as a result of the increased depth of breathing.

## Supporting Information

S1 Supporting Information Supplemental methodological details. (DOCX)
