# Supplementary material for: Parasympathetic Stimuli on Bronchial and Cardiovascular Systems in Humans
Source: PLoS One. 2015 Jun 5;10(6):e0127697. doi: 10.1371/journal.pone.0127697 (PMC4457838; doi:10.1371/journal.pone.0127697)
Supplement: S1 Supporting Information — (DOCX) [file pone.0127697.s001.docx]

**BRONCHIAL AND CARDIOVASCULAR RESPONSES TO PARASYMPATHETIC STIMULI IN HUMANS: PARALLEL OR DIVERGENT?**

**Supplemental Material**

Emanuela Zannin, PhD^1^, Riccardo Pellegrino, MD^2^, Alessandro Di Toro, MD^3^, Andrea Antonelli, MD^2^, Raffaele L Dellacà, PhD^1^, Luciano Bernardi, MD, DMSc^4^

^1^Dipartimento di Elettronica, Informazione e Bioingegneria, Politecnico di Milano, Milano (Italy);

^2^Allergologia e Fisiopatologia Respiratoria, ASO S. Croce e Carle, 12100 Cuneo (Italy);

^3^Dipartimento di Medicina Interna, Università di Pavia e IRCCS Policlinico S. Matteo, Pavia (Italy);

^4^Folkhälsan Institute of Genetics, Folkhälsan Research Center, Biomedicum Helsinki (Finland).

**Address for correspondence:**

Emanuela Zannin

TBM Lab, Dipartimento di Elettronica, Informazione e Bioingegneria

Via G. Colombo 40, 20133 Milano, Italy

Tel. +3902 2399 9025; Fax: +39 02 2399 9000; E-mail: emanuela.zannin@gmail.com

*Pre-study day*

The subjects underwent a spirometric test and methacholine challenge. The former was measured with a mass flow-meter (SensorMedics Inc., CA, USA) following the ATS/ERS recommendations ^1^ . Predicted values are from Quanjer *et al.* ^2^. The bronchial challenge was conducted with MCh chloride dry-powder (Laboratorio Farmaceutico Lofarma, Milan, Italy) dissolved in distilled water and aerosolized by an ampoule-dosimeter system (MB3 MEFAR, Brescia, Italy). The system delivers particles with a median mass diameter of 1.53-1.61μm. The challenge was started with a dose of 300 μg, with doubling increments until the FEV_1_ decreased by 20% or more from control. During aerosol inhalations subjects breathed quietly in a sitting position. Spirometry was measured at baseline and 2 min after each dose of MCh. The provocative dose causing a 20% decrease of FEV_1_ (PD_20_FEV_1_) was calculated by linear interpolation between two adjacent points of the relevant dose-response curves.

*Cardiovascular measurements*

Electrocardiogram was measured by placing three electrodes on the patient's anterior chest wall. Oxygen saturation (SaO_2_) was measured at the finger with a pulse oxymeter (Sat-Trak Pulse Oximeter 767589–103; SensorMedics; Yorba Linda, CA) and expired carbon dioxide (CO_2_) by a capnograph (Cosmo; Novametrix, Wallingford, CT, USA). Tissue oxygen saturation index (TO_2_I) was estimated at the left forearm by a Near Infrared Spectroscope designed, built and validated in our laboratory. Continuous noninvasive arterial blood pressure was monitored (CNAP, CNSsystems, Graz, Austria) via cuffs positioned on the middle finger of the right arm held at the heart level.

*Measurement of airways mechanics*

Airway mechanics was measured by a forced oscillation technique (FOT) previously described ^4–6^ . Sinusoidal pressure oscillations (5, 11, and 19 Hz; ~ 2 cmH_2_O amplitude) were generated by a 16-cm diameter loudspeaker (model CW161N, Ciare, Italy) and applied at the mouth during tidal breathing. The loudspeaker was mounted in a rigid plastic box and connected in parallel to a mesh pneumotachograph and mouthpiece on one side and to a low-resistance high-inertance tube on the other side. Overall load under tidal breathing frequency was 0.98 cm H_2_O·L·s^-1^. Airway opening pressure and flow were recorded by piezoresistive transducers (DCXL10DS and DCXL01DS Sensortechnics, Germany, respectively) and sampled at 200 Hz. A 15 L/min bias flow of air generated by an air pump (CMP08, 3A Health Care, Italy) was used to reduce dead space to about 35 ml. Respiratory resistance was computed by a least squares algorithm ^7,8^ at 5 Hz (R_5_) and 19 Hz (R_19_). Artifacts due to glottis closure or expiratory airflow limitation were avoided by discarding breaths showing any of the following features: i) tidal volume <0.1 L, ii) difference between measured flow oscillation and ideal sine wave with the same Fourier coefficients >0.2 (33), and iii) ratio of minimum to average reactance>3.5 (19). The same breaths were used to measure tidal volume (V_T_) and minute ventilation (V’_E_).

*Computation of baroreflex sensitivity*

Baroreflex sensitivity (BRS) was computed as the mean value obtained from seven different tests ^9^. BRS was first determined from spontaneous fluctuations in the RR interval and SBP during spontaneous and fixed frequency breathing (6 and 15 breath·min^-1^) using the positive and negative sequence methods ^10^, the alpha coefficient in the low and high frequency bands and its average ^11^, and the transfer function technique ^12^. In the subsequent methods, BRS was estimated by identifying spontaneously occurring sequences of 3 or more consecutive heartbeats in which both the SBP and the subsequent RR intervals changed in the same direction. The minimum criteria for change were 1 mm Hg for SBP and 5 ms for the RR intervals. For identified positive and negative sequences with a correlation coefficient between the RR intervals and the SBP exceeding 0.85, the linear regression slopes between SBP and RR intervals were calculated and the average value was taken as a measure of BRS positive and negative slopes, respectively. The alpha coefficient was calculated as the square root of the ratio of the powers of RR intervals and SBP in the low frequency range (0.04–0.15 Hz) and in the respiratory (0.15-0.40Hz) high frequency range when coherence was greater than 0.5 and the phase difference between SBP and RR intervals was negative. In the transfer function method BRS was calculated as the average value of SBP-RR cross-spectrum divided by the SBP spectrum in the low frequency range (0.04–0.15 Hz), when coherence exceeded 0.5. BRS was also obtained by the standard deviation of RR interval divided by the standard deviation of SBP after a high-pass filtering at 0.050 Hz corner frequency, 6dB/octave attenuation, as recently proposed and validated ^13^.

Average HR and SD of each sequence were also calculated.

Root Mean Square of Successive Differences (RMSSD) was computed as the square root of the mean of the squares of the successive differences between adjacent RR intervals.

*Evaluation of the effect of neck suction*

The parasympathetic effects of neck suction were estimated from the power spectra of 0.1Hz spontaneous fluctuations, when the neck suction was timed at 0.1Hz, and from the power spectra at 0.2Hz, when the neck suction was timed at 0.2 Hz. Since breathing frequency was fixed at 15 breath·min^-1^, this approach allowed separating the effects of ventilation, which are more complex than the simple baroreflex effect, from the pure baroreflex stimulus within the respiratory range. To this purpose, coherence between neck suction and RR interval time series in the 0.1 Hz or 0.2 Hz band was tested by using bivariate autoregressive technique. In case coherence was >0.5 then the power in this band was compared with the power in the same band of baseline.

**Reference list**

1. Miller MR, Hankinson J, Brusasco V, et al. Standardisation of spirometry. Rev Mal Respir 2007;24:2S27–S49.

2. Quanjer PH, Tammeling GJ, Cotes JE, Pedersen OF, Peslin R, Yernault JC. Lung volumes and forced ventilatory flows. Eur Respir J [Internet] 1993 [cited 2014 Oct 10];6 Suppl 16:5–40.

3. Dellacà RL, Gobbi A, Pastena M, Pedotti A, Celli B. Home monitoring of within-breath respiratory mechanics by a simple and automatic forced oscillation technique device. Physiol Meas 2010;31:N11–N24.

4. Gobbi A, Milesi I, Govoni L, Pedotti A, Dellaca’ RL. A New Telemedicine System for the Home Monitoring of Lung Function in Patients with Obstructive Respiratory Diseases. 2009 Int Conf eHealth, Telemedicine, Soc Med 2009;117–122.

5. Dellacà RL, Santus P, Aliverti A, et al. Detection of expiratory flow limitation in COPD using the forced oscillation technique. Eur Respir J 2004;23:232–240.

6. Dellacà RL, Pompilio PP, Walker PP, Duffy N, Pedotti A, Calverley PMA. Effect of bronchodilation on expiratory flow limitation and resting lung mechanics in COPD. Eur Respir J 2009;33(6):1329–37.

7. Kaczka DW, Barnas GM, Suki B, Lutchen KR. Assessment of time-domain analyses for estimation of low-frequency respiratory mechanical properties and impedance spectra. Ann Biomed Eng;23(2):135–51.

8. Kaczka DW, Ingenito EP, Lutchen KR. Technique to determine inspiratory impedance during mechanical ventilation: implications for flow limited patients. Ann Biomed Eng;27(3):340–55.

9. Bernardi L, Barbieri G De, Rosengård-Bärlund M, Mäkinen V-P, Porta C, Groop P-H. New method to measure and improve consistency of baroreflex sensitivity values. Clin Auton Res 2010;20:353–361.

10. Bertinieri G, Rienzo M di, Cavallazzi A, Ferrari AU, Pedotti A, Mancia G. A new approach to analysis of the arterial baroreflex. J Hypertens Suppl 1985;3:S79–S81.

11. Pagani M, Somers V, Furlan R, et al. Changes in autonomic regulation induced by physical training in mild hypertension. 1988.

12. Pinna GD, Maestri R. Reliability of transfer function estimates in cardiovascular variability analysis. Med Biol Eng Comput 2001;39:338–347.

13. Mirizzi G, Giannoni A, Bramanti F, et al. A simple method for measuring baroreflex sensitivity holds prognostic value in heart failure. Int J Cardiol 2013;169(1):e9–11.
